# Experimental investigation on mechanical properties and strength criteria of frozen soft rock

**DOI:** 10.1371/journal.pone.0313493

**Published:** 2025-01-10

**Authors:** Zhenhua Wang, Zecheng Wang, Dongwei Li, Zhiwen Jia, Xiqi Liu

**Affiliations:** 1 School of Civil and Architectural Engineering, East China University of Technology, Nanchang, China; 2 College of Civil Engineering and Architecture, Dalian University, Dalian, China; 3 Pearl River Water Resources Research Institute, Guangzhou, China; Islamic Azad University Mashhad Branch, ISLAMIC REPUBLIC OF IRAN

## Abstract

Excavation of underground engineering structures involving deeply buried water-rich soft rocks is generally carried out using the artificial freezing method. A series of undrained uniaxial and triaxial shear and creep tests were conducted on soft rocks under different confining pressures (0, 0.2, 0.5, and 1.0 MPa) at different freezing temperatures (room temperature, -5°C, -10°C, and -15°C). Test results indicate that the frozen soft rocks show strain softening characteristics. The stress—strain curve changes from a straight line to a curve as deviatoric stress constantly increases, while it decreases abruptly after the deviatoric stress reaches the peak and is slightly affected by the freezing temperature. At the same temperature, shear strength increases at a rate of 5.6 MPa/°C with increasing confining pressure; as freezing temperature decreases, the shear strength increases at 0.34 MPa/°C, and cohesion increases at 0.6 MPa/°C. Under the same confining pressure, the failure strain of soft rock decreases with the decrease of temperature. The Mohr-Coulomb (M-C) criterion can accurately describe the failure process of frozen soft rocks in the pre-peak stage, with a correlation coefficient greater than 0.98. Within the test stress range, soft rocks display attenuated stable creep deformation. Acoustic emission (AE) tests were conducted to further verify that the soft rocks show shear failure under load, with a shear plane showing an angle of 45° with the horizontal. The research findings provide technical support and theoretical reference for studying rock mechanical properties as well as for designing and carrying out underground freezing of rocks in a low-temperature environment.

## 1. Introduction

The artificial freezing method consists of freezing water in rocks to be excavated through artificial refrigeration, forming a closed freezing curtain. Excavation and construction can be performed under the protection of the freezing curtain, which seals off water and provides temporary support [1–3]. The artificial freezing method has been extensively used to reinforce water-rich underground engineering strata, including subway tunnels, deep foundation pits, and bridge pier foundations [4–6]. It has wide applications in underground engineering structures involving deep rocks, such as deep mine shafts, diversion tunnels, and underground energy engineering [7–11].

Scholars in China and abroad have carried out numerous studies on the mechanical properties of frozen rocks. For example, by performing triaxial creep tests under different confining pressures and low-temperature freezing conditions. Wei et al. [12] analyzed the influences and damage effects of temperature on mechanical properties of the samples as well as developed a creep constitutive model. They found that the model highly tallies with the test results. Taking freeze-thaw cycles and confining pressure as variables. Zhang et al. [13] tested the mechanical properties of rock samples under various factors and established a damage model for rocks, which describes the rock deformation process. Zhu et al. [14] carried out triaxial compression tests to investigate changes in the peak strength and elastic modulus of rock samples under various temperatures and confining pressures. By performing laboratory model tests. Rong et al. [15] studied the formation mechanism of artificially frozen walls in large-flow-rate permeable strata. Chen et al. [16] performed cyclic freeze-thaw tests and simulations to analyze the key factors influencing the strength of low-temperature rock samples. Li et al. [17] studied the fatigue strength of frozen sandstone using creep tests and summarized the failure law. Li [18] carried out multi-level loading and unloading creep tests considering different water contents and established a corresponding creep model. Yang et al. [19] studied correlations between the creep properties of fractured hard rocks and intact rocks. For very soft coal rocks, Wang et al. [20] conducted uniaxial and triaxial creep tests under single-level load using a self-developed long-term triaxial creep testing system as well as examined the long-term creep properties of very soft coal rocks. Through compression creep tests on soft rocks, Miao et al. [21] explored the large-creep deformation law of soft rocks in high and steep slopes of open pit mines as well as established the creep-large deformation constitutive model for soft rocks.

By conducting laboratory tests on rockburst likelihood and the acoustic emission (AE) test under uniaxial compression on limestone of different burial depths, Wang et al. [22] analyzed the AE characteristics of limestone of different rockburst likelihood levels. Based on the basic principles of damage mechanics, they also established a damage model characterized by the accumulated AE energy. For red-bed soft rocks eroded by chemical solutions with varying pH values, Li et al. [23] analyzed the damage and failure process under uniaxial compression using the AE technology.

Despite the fruitful achievements obtained by numerous scholars through in-depth research on the mechanical properties of multiple types of frozen soft rocks, the mechanical properties and strength criteria of frozen soft rocks remain poorly studied. Undrained uniaxial and triaxial shear and creep tests as well as AE tests were conducted on soft rocks under different confining pressures and freezing temperatures. The strength criteria and creep laws of frozen soft rocks were obtained. Also, the findings of this study provide technical support and theoretical reference for future studies on the mechanical properties of and for designing and carrying out underground freezing of rocks at low temperature.

## 2. Test samples and schemes

**Fig 1:**
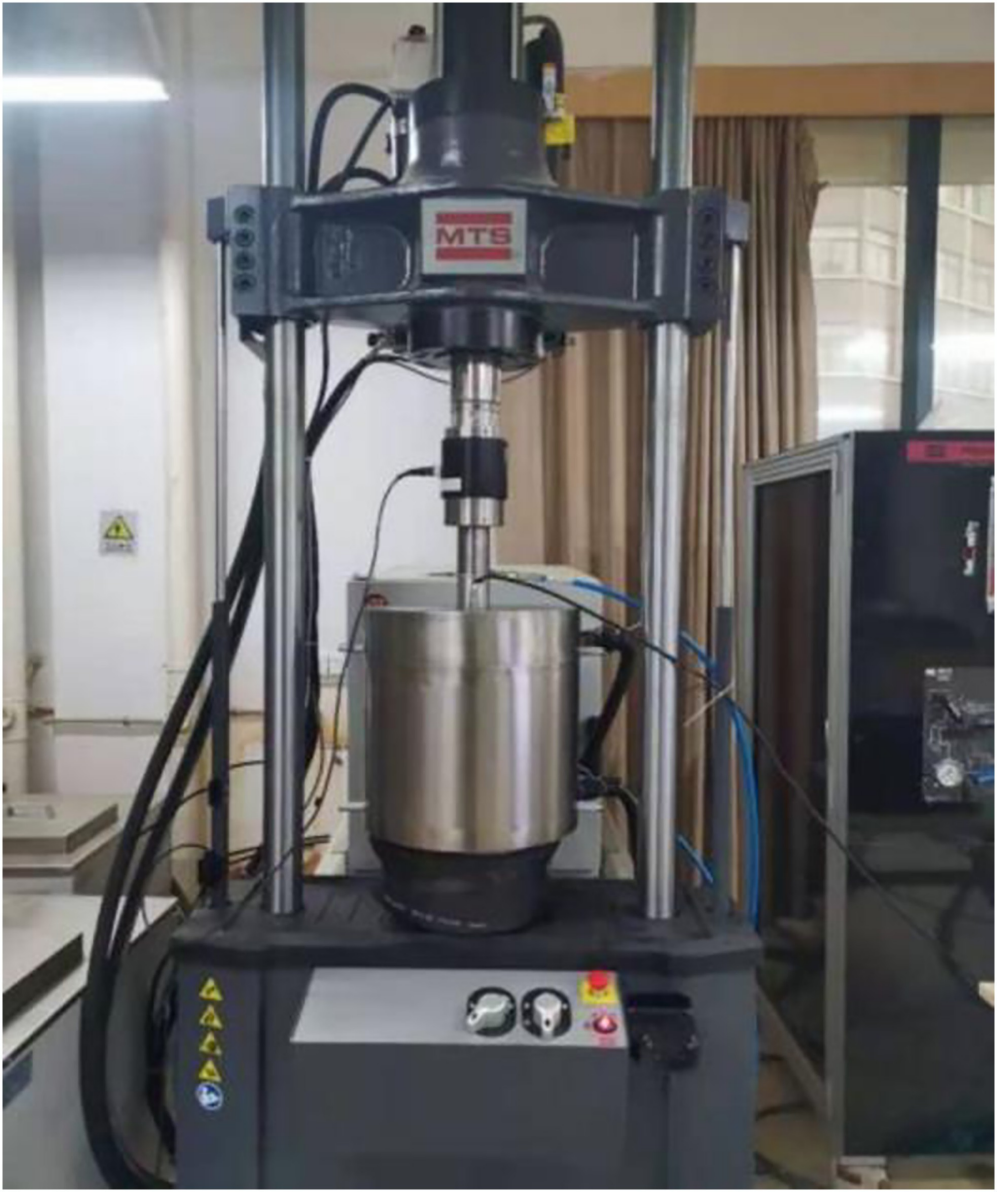
MTS-370.25 fatigue resting system.

Tests on soft rocks were conducted using an MTS triaxial testing system for frozen soils as shown in Fig 1. Four confining pressures were set according to the burial depths of surrounding rocks, that is, 0, 0.2, 0.5, and 1.0 MPa. The corresponding sampling depths were about 0 m, 75 m, 190 m and 380 m, respectively. To carry out experimental research on the uniaxial strength, triaxial shear strength, and creep properties of rocks under different confining pressures and freezing temperatures (-5, -10, and -15°C). These three temperatures are often studied in frozen soil mechanics, especially-10°C must be included in frozen soil mechanics.

**Fig 2:**
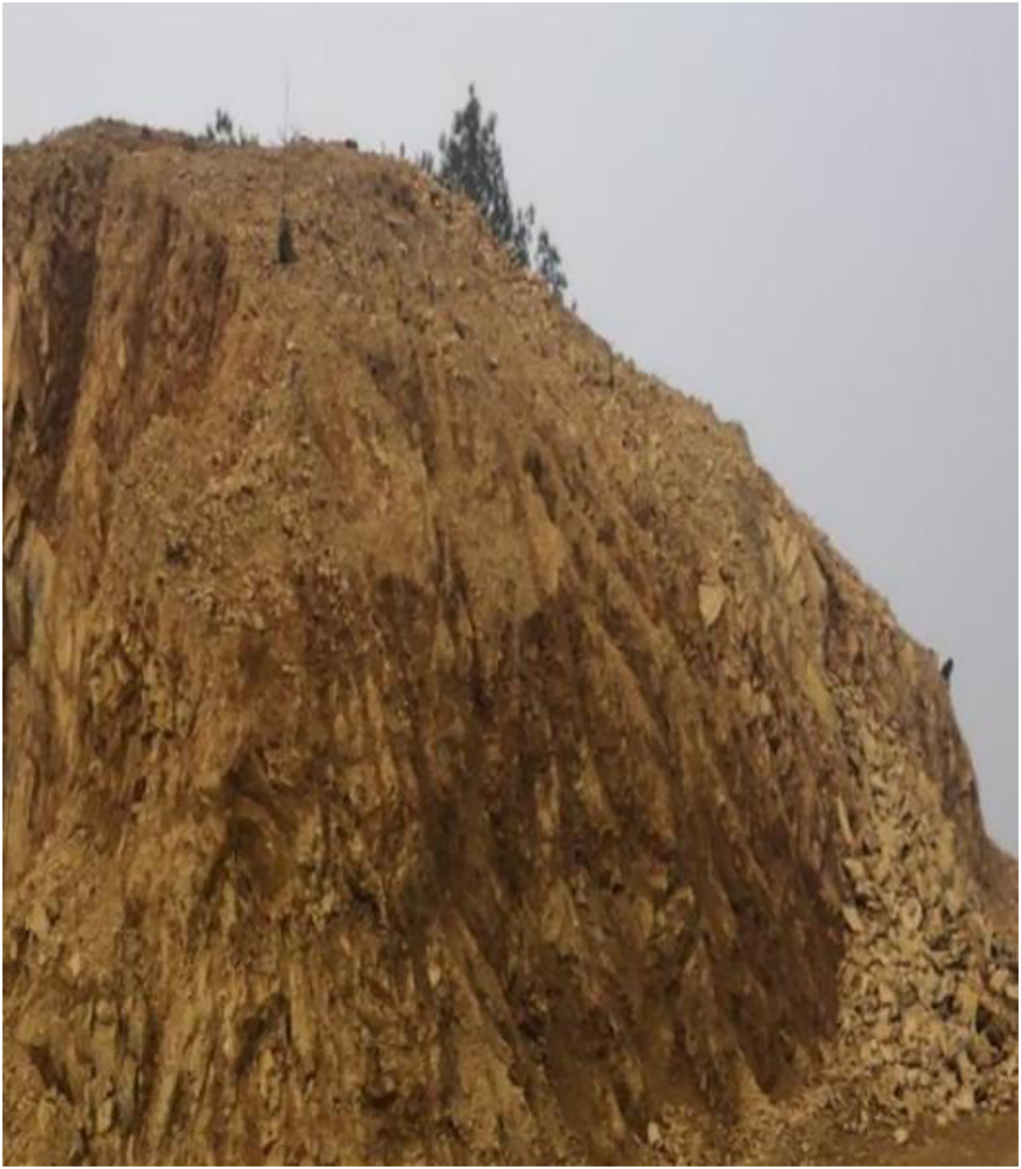
Sampling site.

Test samples were composed of soft rocks collected from the third-phase project of diversion tunnels in a city, as shown in Fig 2. The soft rocks were first machined into cylindrical samples measuring Φ50 mm × 100 mm and then cured for 24 h at -5, -10, and -15°C. The initial moisture content of the sample is fixed at 2%. The displacement-controlled loading mode was used in the triaxial shear test with a displacement rate of 1 mm/min. According to the standards in Artificial Frozen Soil Physics Mechanics Performance Test (MT/T593.6–2011), the test was stopped when the axial strain reached to 20% or the peak stress declined by 20%.

The creep test employed three levels of stress, specifically n times the shear strength, with n values of 0.3, 0.5, and 0.7. The test was stopped when the deformation of the samples remained stable for 12 h or the samples were damaged. The specific test schemes are given in Table 1.

**Table 1:** Test schemes for soft rocks.

Sample number	Confining pressure (MPa)	Temperature (°C)	Test
**DJ-1**	0	25	Uniaxial shear test
**DR-2**			Three-level uniaxial creep test
**SR-1**	0.2		Three-level triaxial creep tests
**SR-2**	0.5		
**SR-3**	1.0		
**SJ-1**	0.2		Triaxial shear test
**SJ-2**	0.5		
**SJ-3**	1.0		
**SJ-4**	0.2	-5	
**SJ-5**	0.5		
**SJ-6**	1.0		
**SJ-7**	0.2	-10	
**SJ-8**	0.5		
**SJ-9**	1.0		
**SJ-10**	0.2	-15	
**SJ-11**	0.5		
**SJ-12**	1.0		

## 3. Analysis of mechanical properties

Mechanical properties are key indicators for evaluating the reliability and durability of materials. Uniaxial, triaxial, and acoustic emission tests are performed on the samples. A series of analyses reveal the response mechanisms, strength characteristics, and potential failure modes of the materials under different load conditions.

### 3.1 Stress—strain relationship

**Fig 3:**
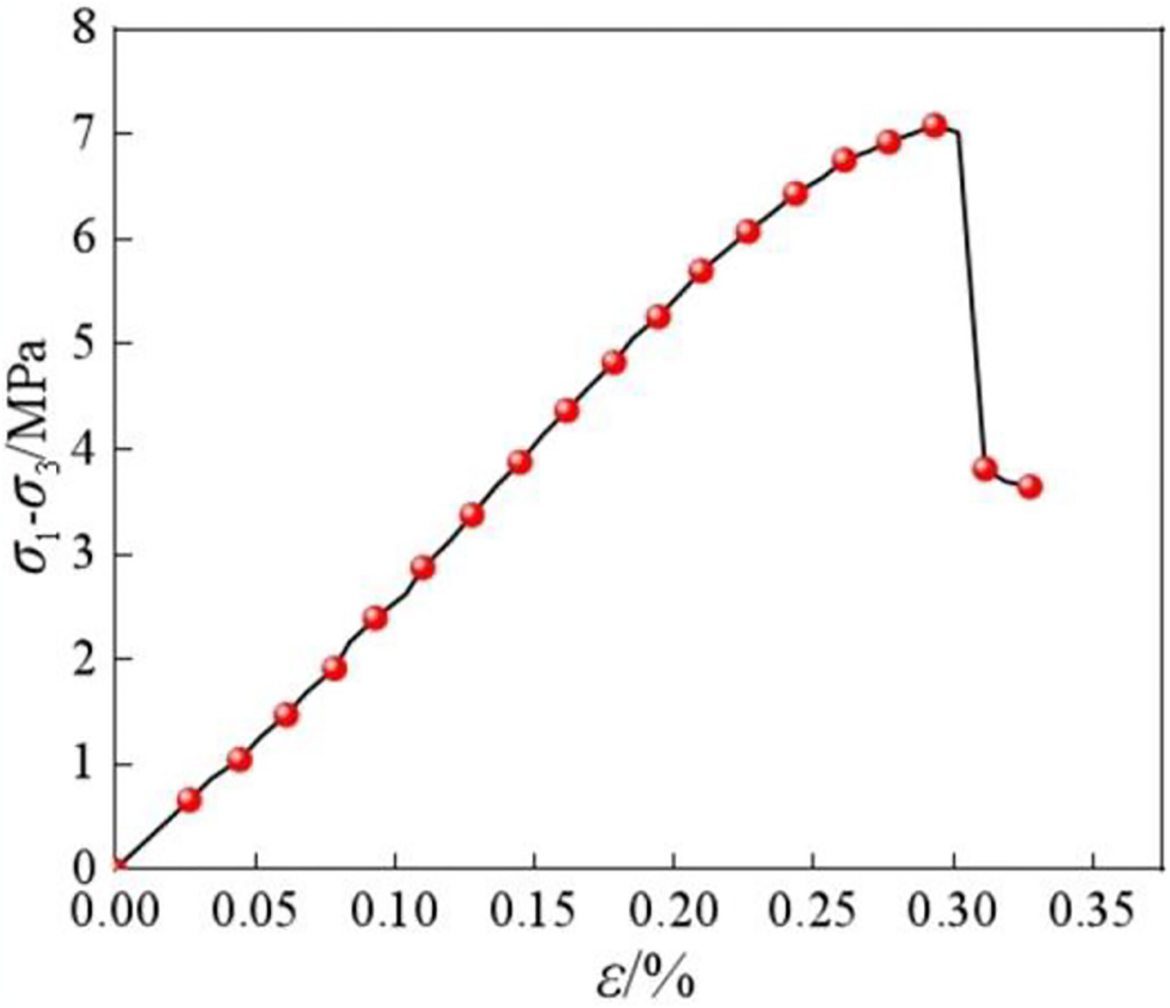
Uniaxial shear curve of soft rocks at room temperature.

Fig 3 illustrates the stress—strain curve for the uniaxial compression test at room temperature. The uniaxial compressive strength linearly grows with increasing strain first and then abruptly decreases after reaching the peak stress. The frozen soft rocks show a clear brittle failure mode, with strain softening characteristics.

**Fig 4:**
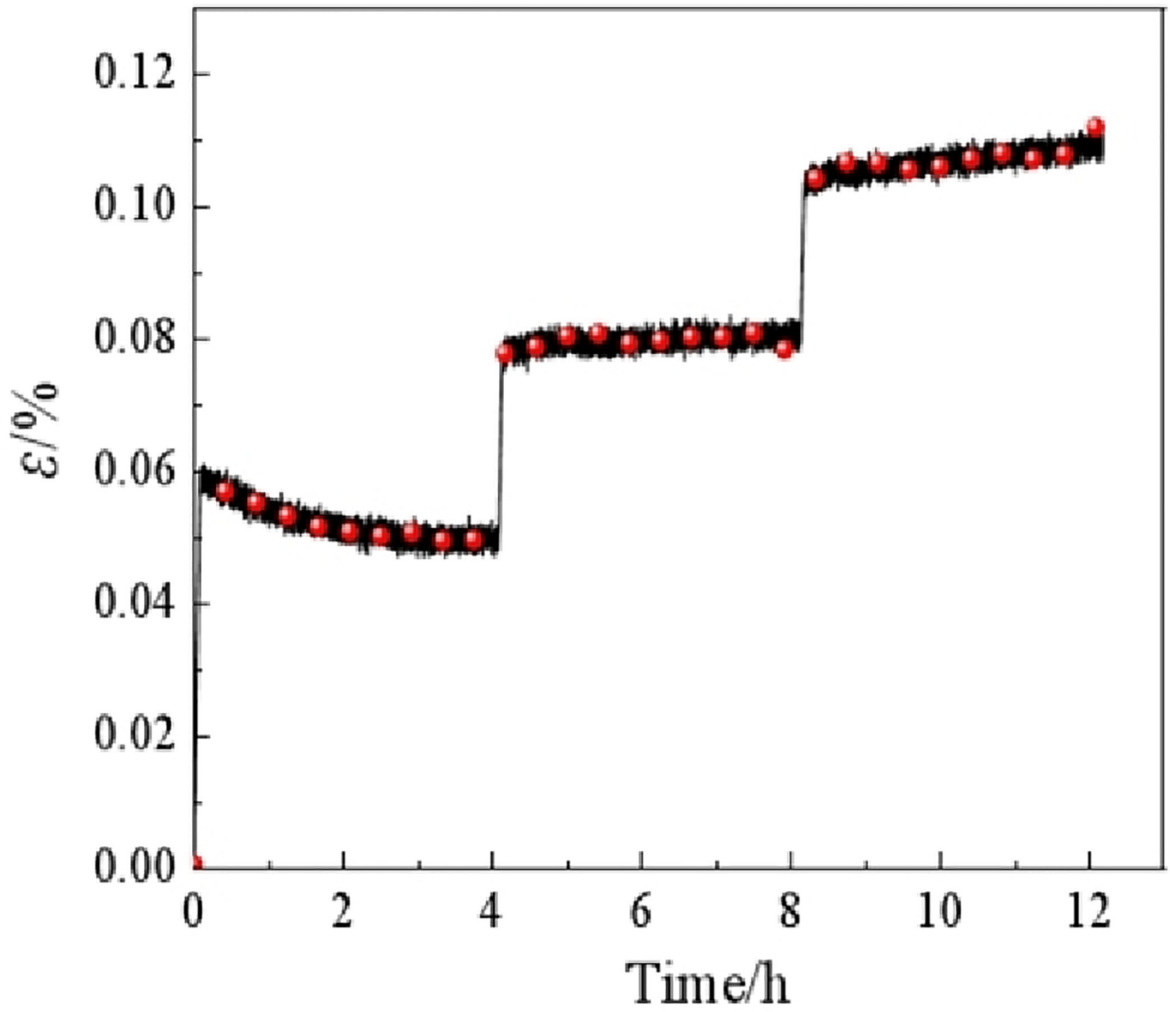
Three-level uniaxial creep curve of soft rocks at room temperature.

Fig 4 shows the three-level uniaxial creep curve of soft rocks. As the stress increases to the pre-set value, the displacement of soft rocks varies abruptly, as evinced by the sharp ascent of the curve with an angle 90°. The displacement changes little with time after reaching the pre-set stress, and the curve forms a step-like shape. The strain at the first level decreases slightly, while it reaches a maximum and stabilizes in the latter two levels. The three-level uniaxial creep curve of soft rocks is shown as an attenuated stable creep curve overall.

**Fig 5:**
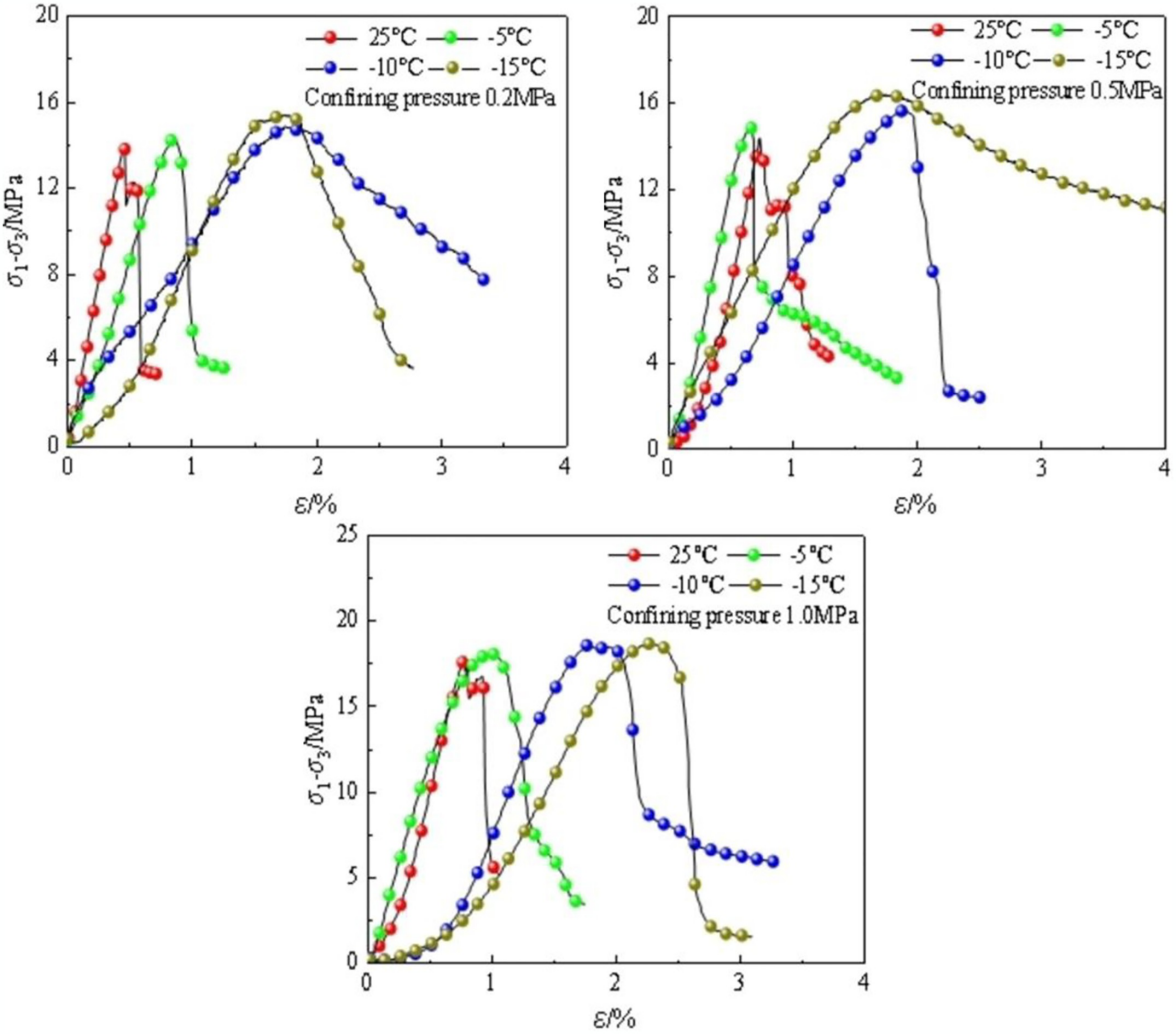
Stress—strain curves under different temperatures and confining pressures.

The stress—strain curves of frozen soft rocks in the uniaxial and triaxial compression tests at different temperatures are illustrated in Fig 5. The uniaxial compression and triaxial compression can both be divided into three stages, including a linear elastic stage, yield stage, and failure stage. As freezing temperature decreases, the strength of soft rocks increases with an incremental increase in confining pressure. Under the same confining pressure, the failure strain of the soft rocks decreases with decreasing temperature, showing brittle failure characteristics.

**Fig 6:**
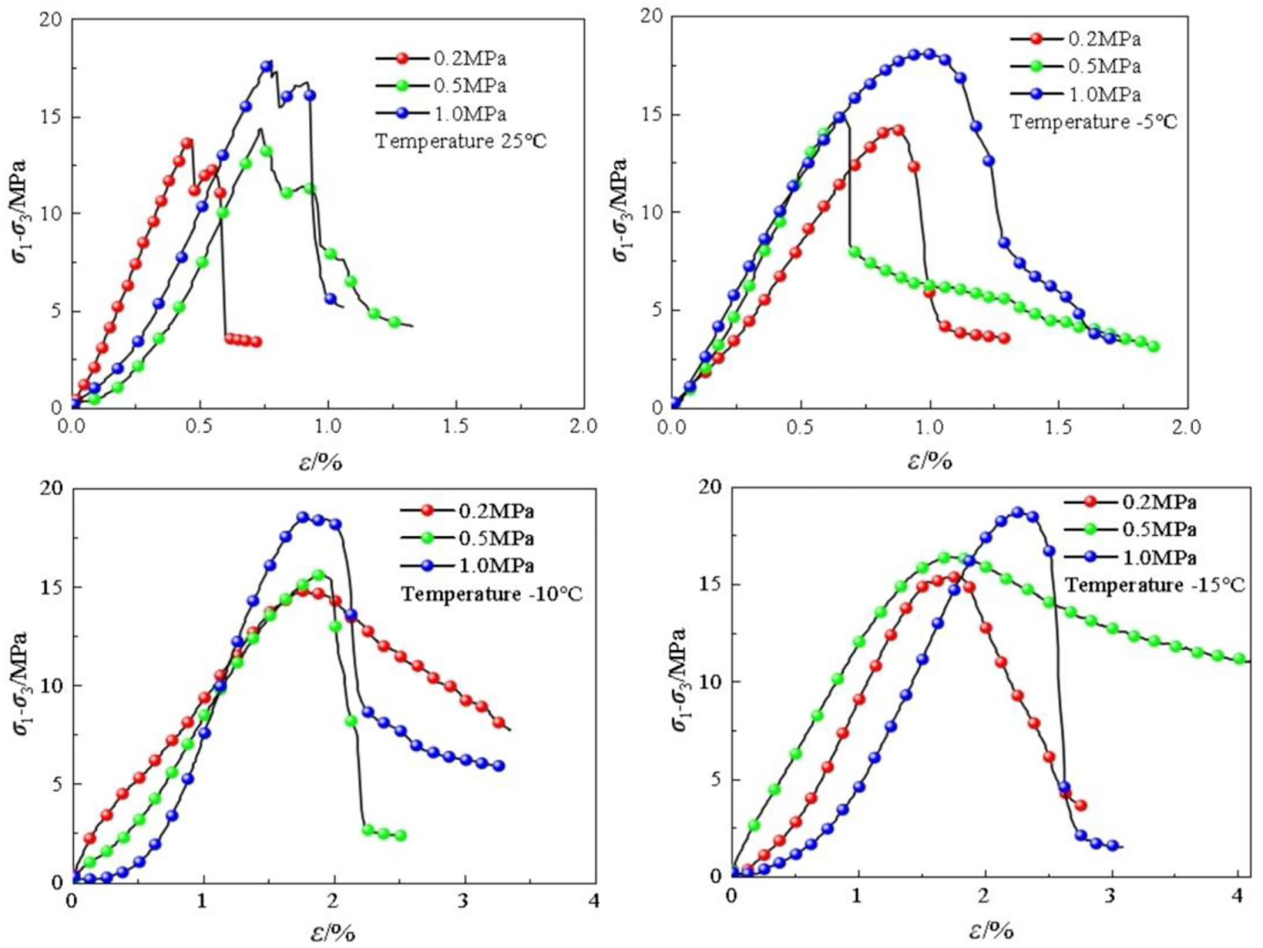
Stress-strain curves at different temperatures and confining pressures.

Fig 6 depict the triaxial test results under different confining pressures and freezing temperatures. There are two peaks on the curve at room temperature: after the occurrence of the first peak, the second peak appears after a 0.2% increase in strain. Another peak appears because cracks in the soft rocks are compacted after the first peak, increasing the bearing capacity of rock. The frozen soft rocks undergo brittle failure, and the stress—strain curves display softening characteristics. At a freezing temperature of -10°C, the strength, elastic modulus, and Poisson’s ratio of the frozen soft rocks are 17.5 MPa, 85 MPa, and 0.25, respectively. For the same temperature, the shear strength grows at a rate of 5.6 MPa/°C with increasing confining pressure; the shear strength decreases with increasing freezing temperature at a rate of 0.34 MPa/°C. With decreasing freezing temperature, cohesion grows at 0.6 MPa/°C, and the internal friction angle gradually decreases.

**Fig 7:**
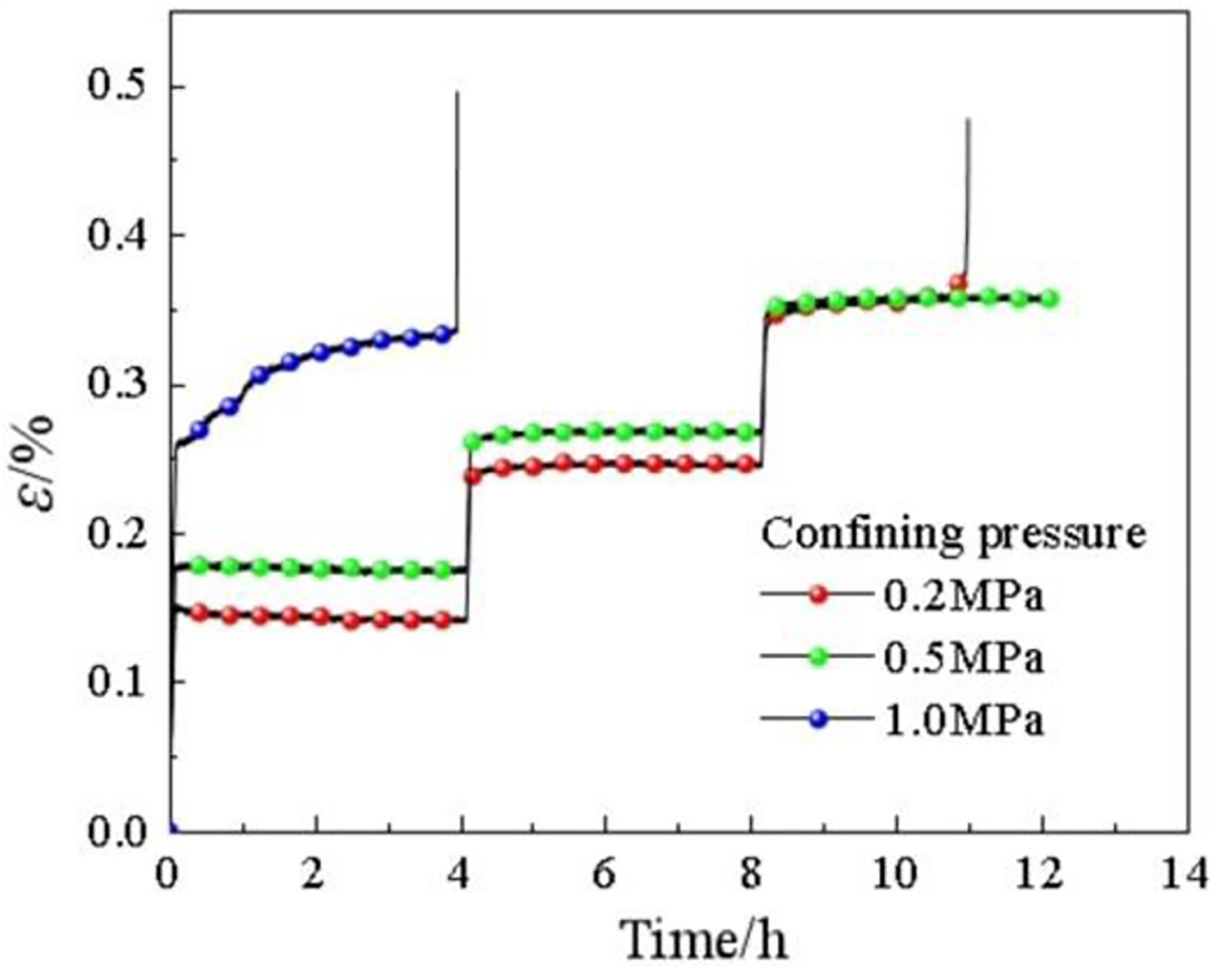
Three-level creep curves of soft rocks at room temperature under different confining pressures.

By carrying out triaxial creep tests at room temperature, creep curves of soft rocks were obtained as shown in Fig 7. A single sample was subjected to three-level loading under creep coefficients of 0.3, 0.5, and 0.7. Results show that the creep changes rapidly, as evinced by the curve ascending with an angle of 90° when the stress increases to the pre-set value. After reaching the pre-set stress value, the displacement of soft rocks varies slightly with time, and the curves are step-like in shape. The creep curve at the third level is incomplete under 0.2 MPa, implying destructive creep; the creep curve is not damaged under 0.5 MPa, indicating stable creep; the curve at the second level is also incomplete under 1.0 MPa, also showing destructive creep.

**Fig 8:**
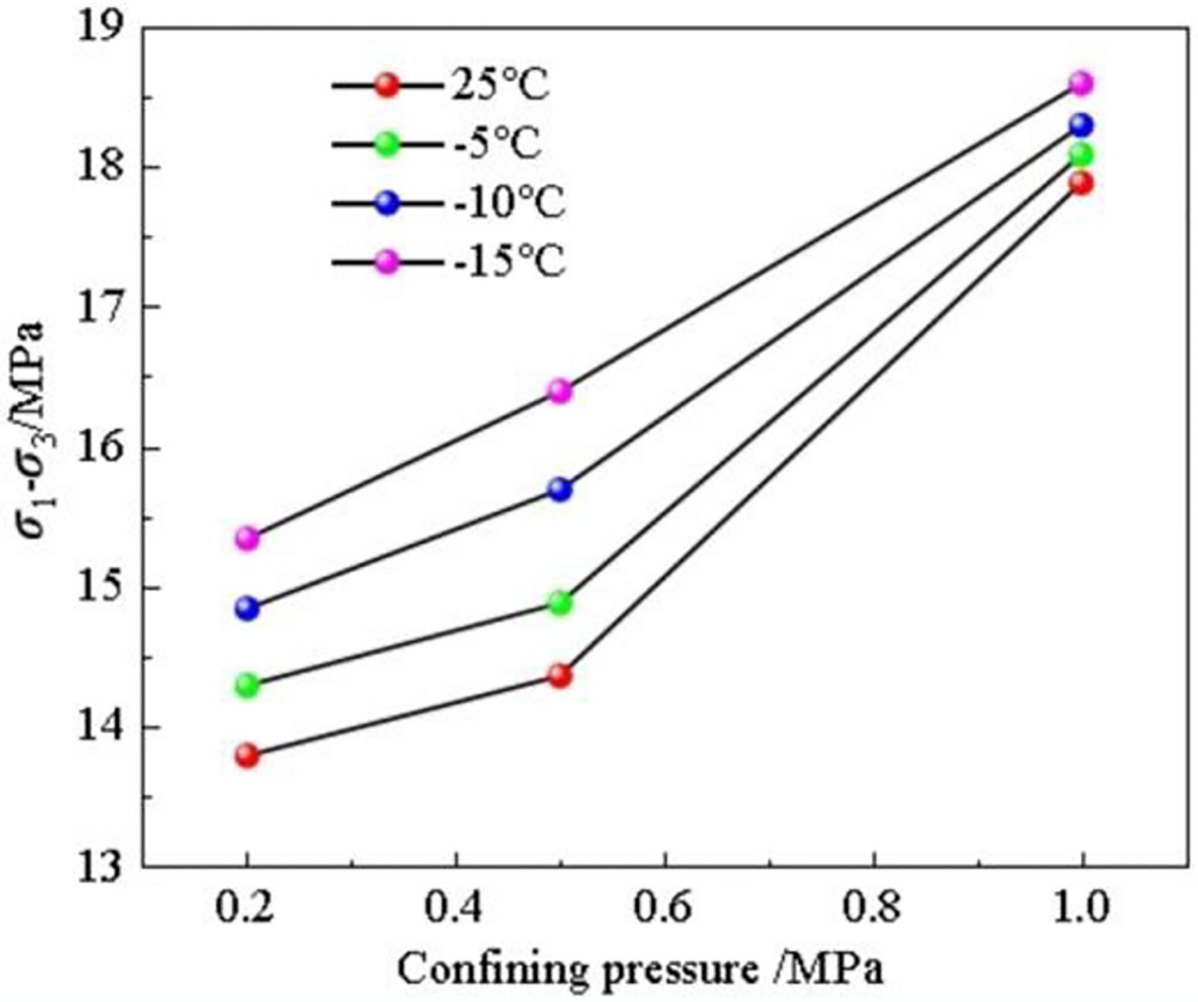
Maximum shear strength of soft rocks under different confining pressures and freezing temperatures.

By summarizing the shear strength of rocks under different confining pressures and freezing temperatures, the shear strength of the soft rocks gradually grows with decreasing freezing temperature and rising confining pressure as shown in Fig 8. The intensity change amplitude increases with the decrease of freezing temperature and the increase of confining pressure.

### 3.2 Failure criteria

**Fig 9:**
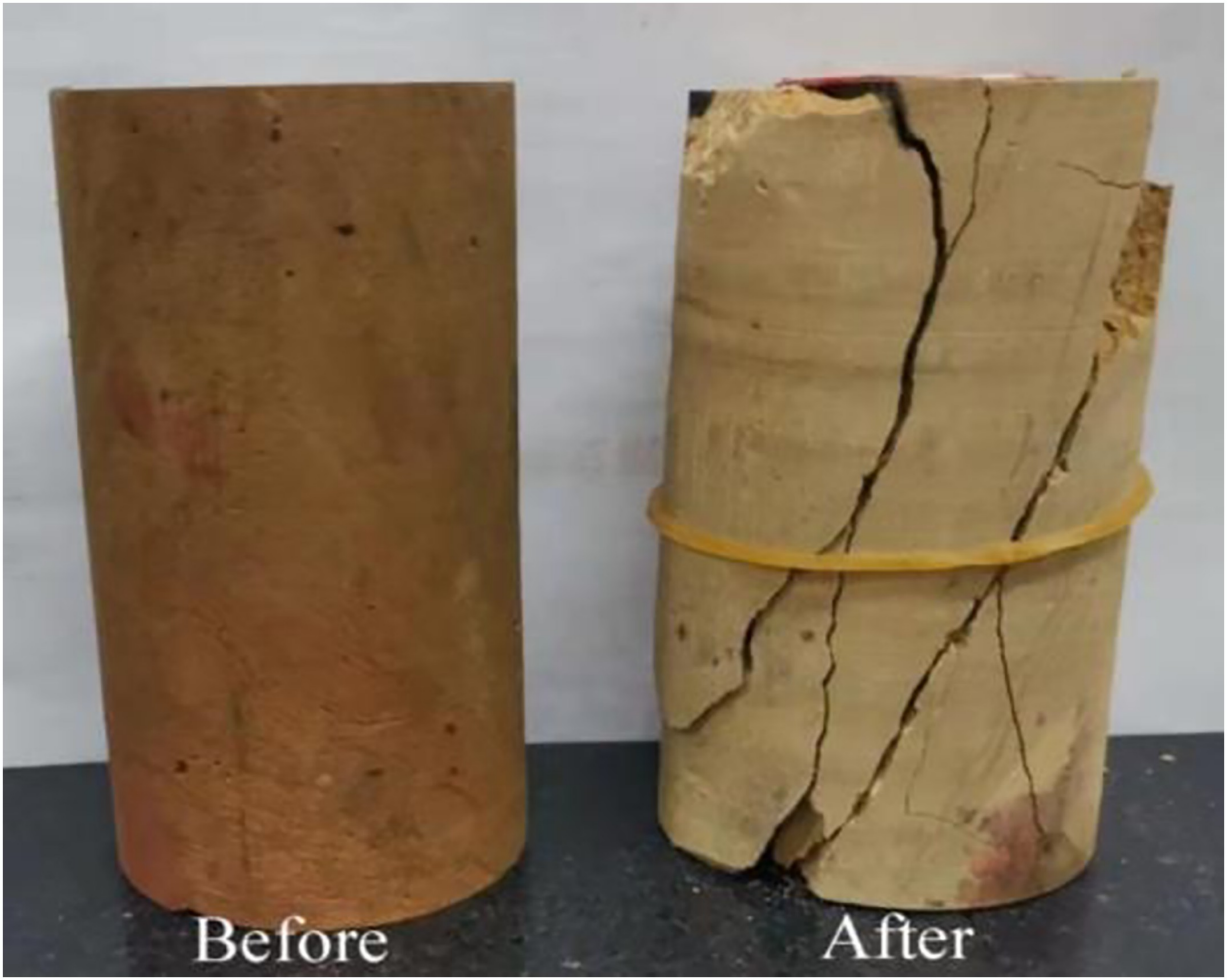
Failure mode of the sample.

Triaxial compression tests were carried out on soft rocks at different temperatures under different confining pressures based on the above test schemes. The failure mode of the samples is shown in Fig 9. The shear plane has an angle of 45° with the horizontal, and the sample exhibits shear failure.

The general form of the Mohr-Coulomb (M-C) strength criterion is:

$$\tau_{f}(T)=\sigma \text{tan}\varphi \left(T\right)+c\left(T\right)$$ (1)

where *τ*_*f*_(*T*) and σ refer to the shear strength of frozen soft rocks (MPa) and the normal stress on the shear plane (MPa), respectively; *φ*(T) and *c*(T) represent the internal friction angle (°) and cohesion (MPa), respectively, both of which are related to the freezing temperature.


$$\sigma =\frac{1}{2}\left(\sigma_{1}+\sigma_{3}\right)+\frac{1}{2}\left(\sigma_{1}-\sigma_{3}\right)\text{cos}2\alpha$$ (2)

$$\tau =\frac{1}{2}\left(\sigma_{1}-\sigma_{3}\right)\text{sin}2\alpha$$ (3)

Where $\alpha =45{}^{\circ}+\frac{\varphi }{2}$.

After the soil is loaded, the normal stress on any surface is supported by the solid particle skeleton and the pore water or gas. That is,

σ‘=σ−μ (4)

Where *σ‘* represent the effective stress; *μ* represent the pore pressure.

**Fig 10:**
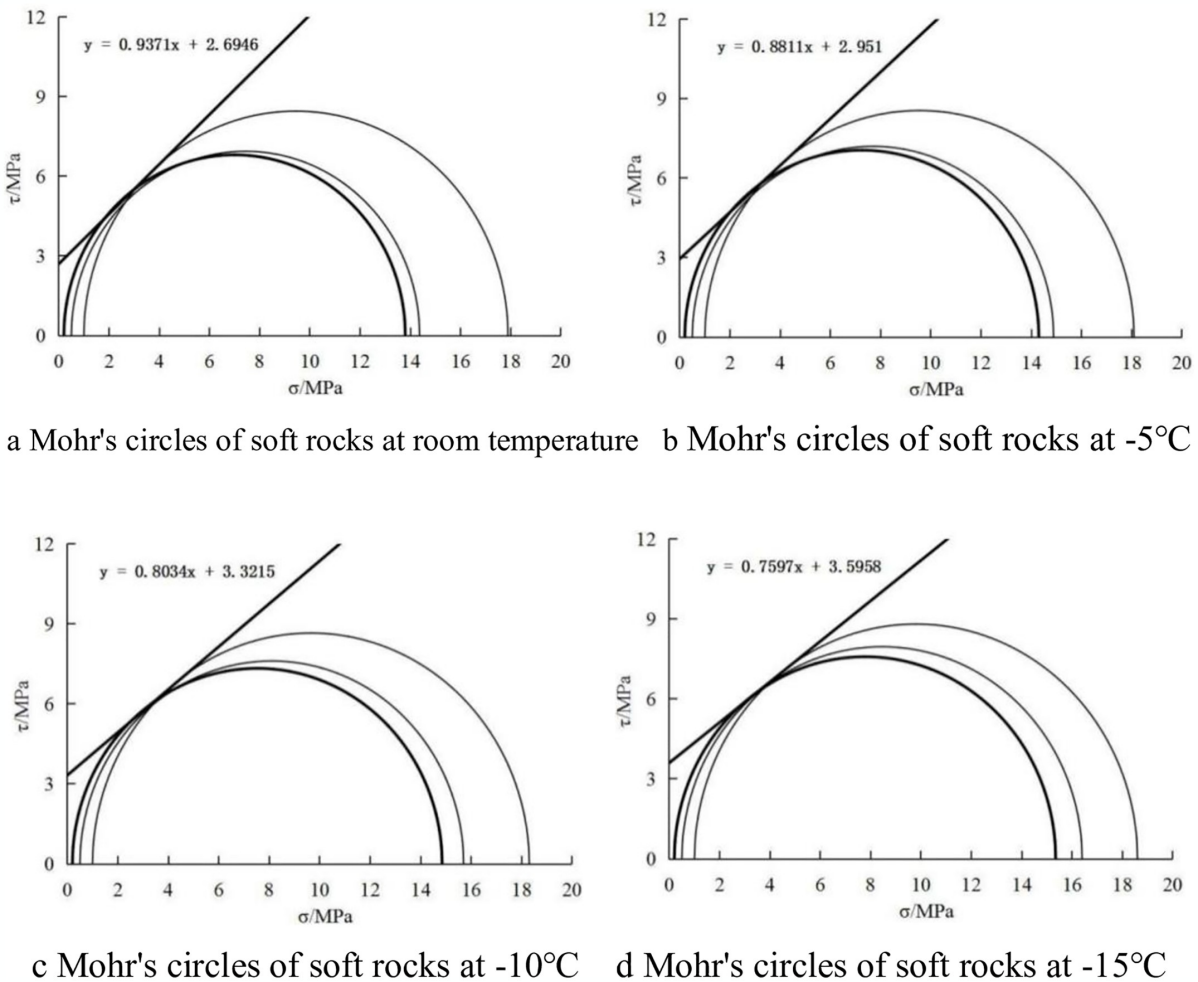
Mohr’s circles of soft rocks at different temperatures. (a) Mohr’s circles of soft rocks at room temperature. (b) Mohr’s circles of soft rocks at -5°C. (c) Mohr’s circles of soft rocks at -10°C. (d) Mohr’s circles of soft rocks at -15°C.

Three sets of triaxial tests were conducted on frozen soft rock, each subjected to distinct confining pressures of 0.2 MPa, 0.5 MPa, and 1.0 MPa. Based on the project’s specific requirements, an axial strain of 20% was designated as the failure criterion, with the corresponding deviatoric stress (*σ*_1_-*σ*_3_) serving as the failure strength indicator. Utilizing the *σ*_1_f-*σ*_3f_/2 values obtained under various confining pressures as radii, molar circles were plotted, as illustrated in Fig 10(a)–10(d).

Fig 10 shows the Mohr’s circles of soft rocks at different temperatures. At -10°C, the strength, elastic modulus, and Poisson’s ratio of frozen soft rocks are 17.5 MPa, 85 MPa, and 0.25, respectively. The failure process of the frozen soft rocks can be described using the M-C criterion, with a correlation coefficient greater than 0.98. When delineating the failure mechanism of soft rock, the criterion offers a mechanical model grounded in two pivotal parameters: cohesion (*c*) and the internal friction angle (*φ*). In soft rock, the inherent inhomogeneity and the presence of cracks often lead to localized stress concentrations. Consequently, soft rock is particularly susceptible to shear failure under shear stress conditions, particularly when the Mohr stress circle aligns tangentially with the Coulomb shear strength line.

### 3.3 AE tests

**Fig 11:**
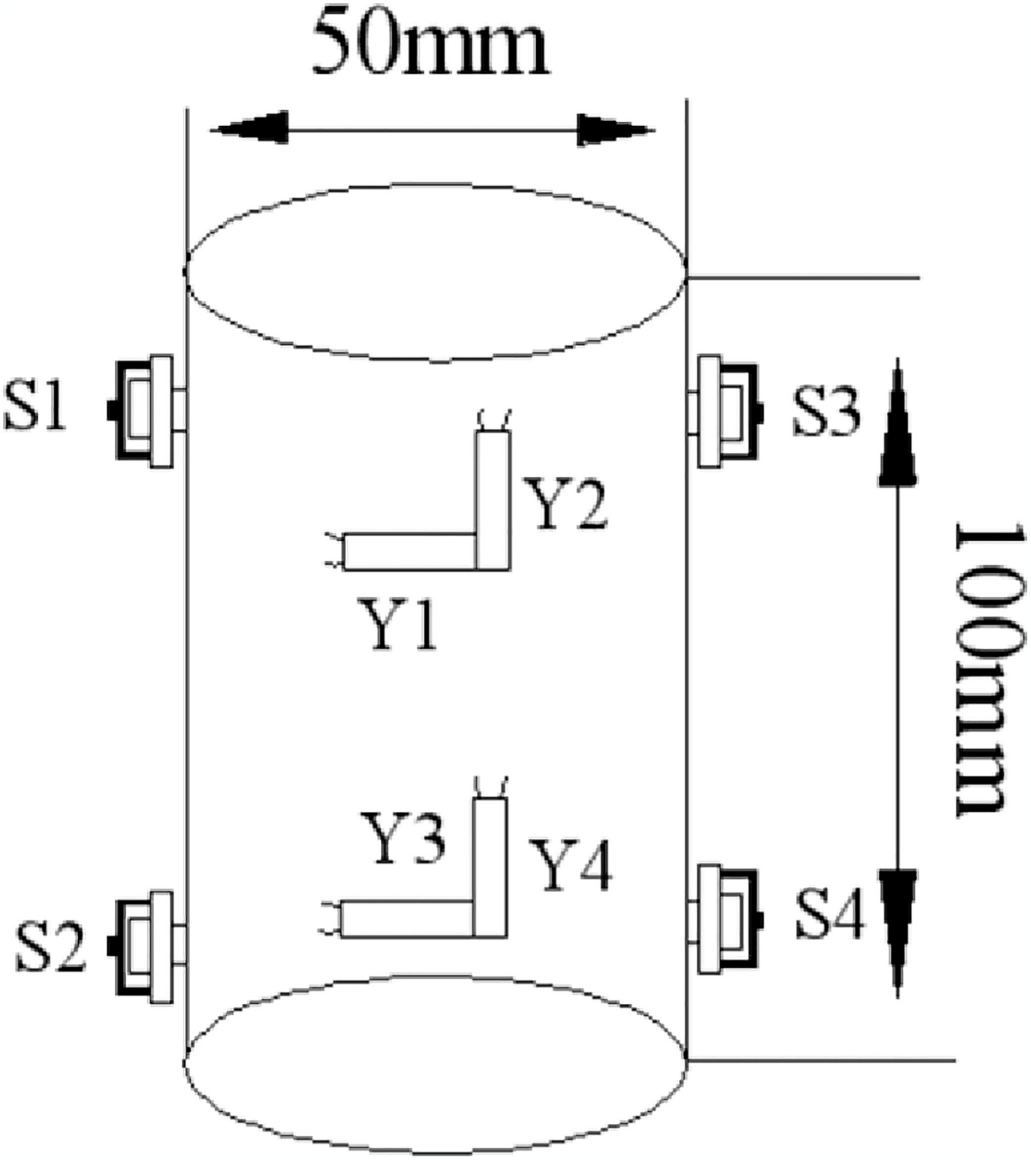
Positions of AE probes and strain gauges.

**Fig 12:**
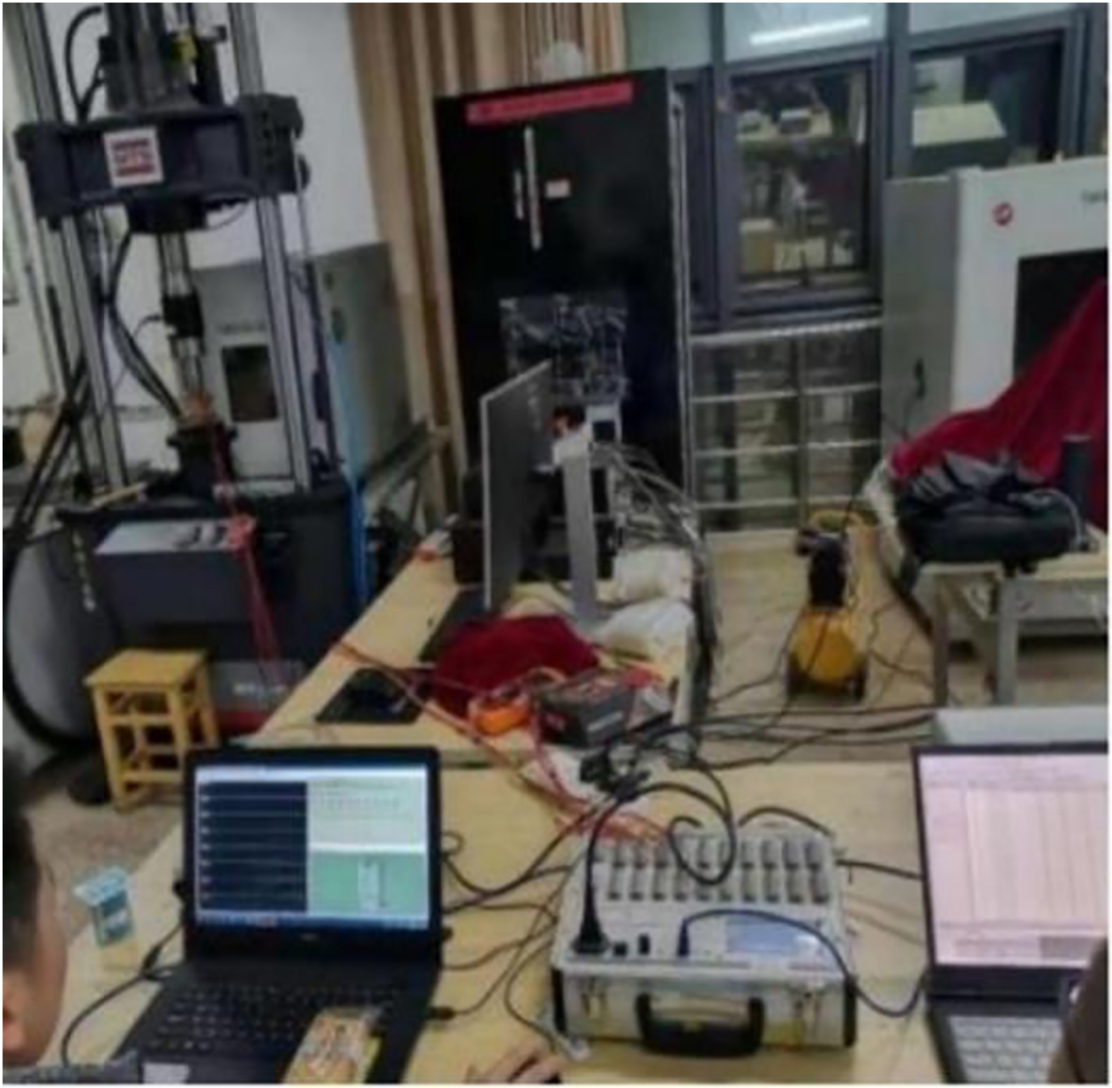
AE monitoring system.

**Fig 13:**
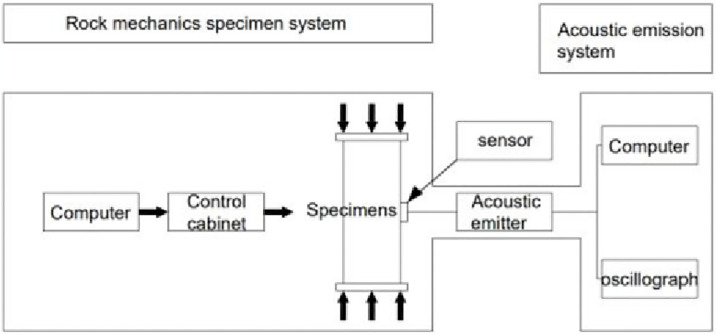
Schematic diagram of the AE testing system.

**Fig 14:**
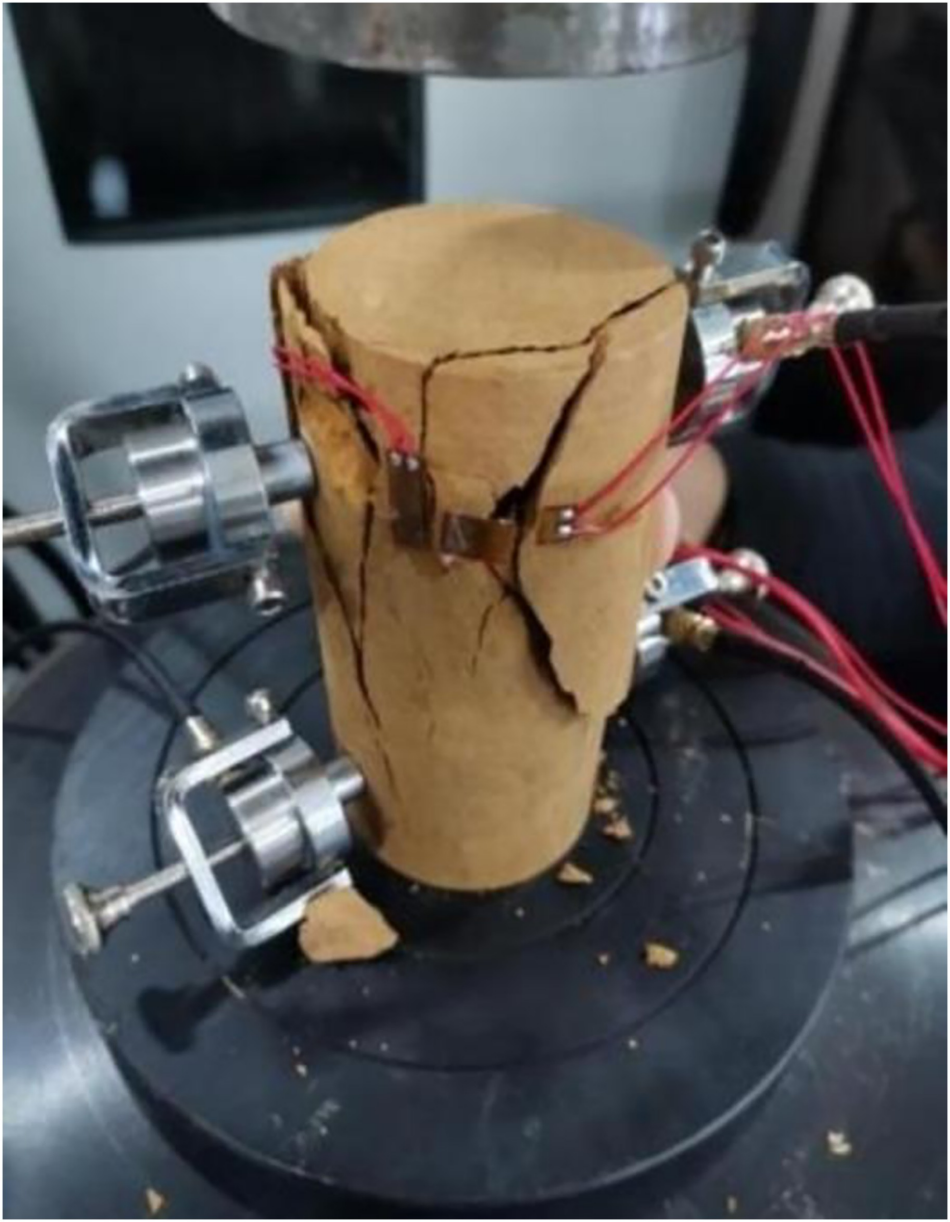
Failure mode of the sample.

Acoustic emission technology can comprehensively and accurately reflect the fracture characteristics of rocks [24, 25]. AE tests were carried out to obtain the failure characteristics of frozen soft rocks by attaching four AE sensors and four strain gauges on the gummed samples. While performing uniaxial compression tests, AE and strain data were collected at four points using data collectors. The positions of each strain gauge and AE sensor are shown in Fig 11. The test equipment was primarily composed of an MTS testing system, an AE system, a data acquisition system for strain gauges, and other measurement components. The test site and the system are separately shown in Figs 12 and 13. The sample was damaged by a shear plane with an angle of 45° with the horizontal, some AE sensors fell off, and some strain gauges were fractured at the end of the test, as shown in Fig 14. When an axial compressive stress is applied to the sample, because the transverse tensile stress exceeds the ultimate tensile strength of the rock, transverse tensile strain occurs in the sample, which marked by clear volume expansion as well as a few fragments falling off during loading.

**Fig 15:**
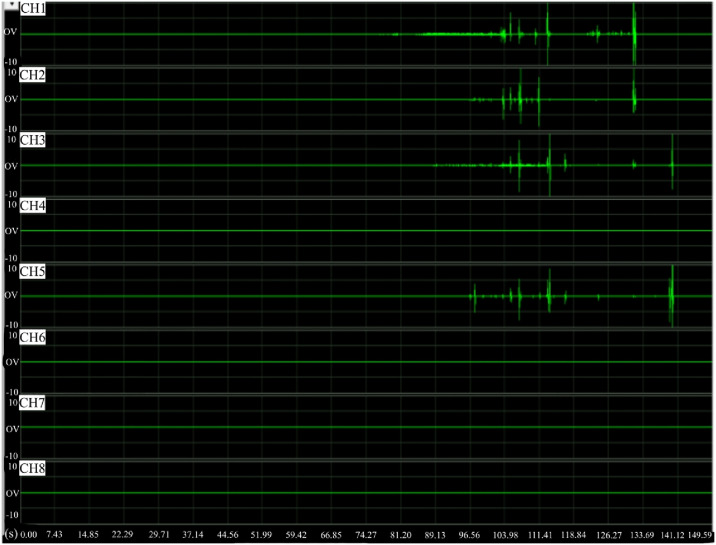
Received AE waves.

**Fig 16:**
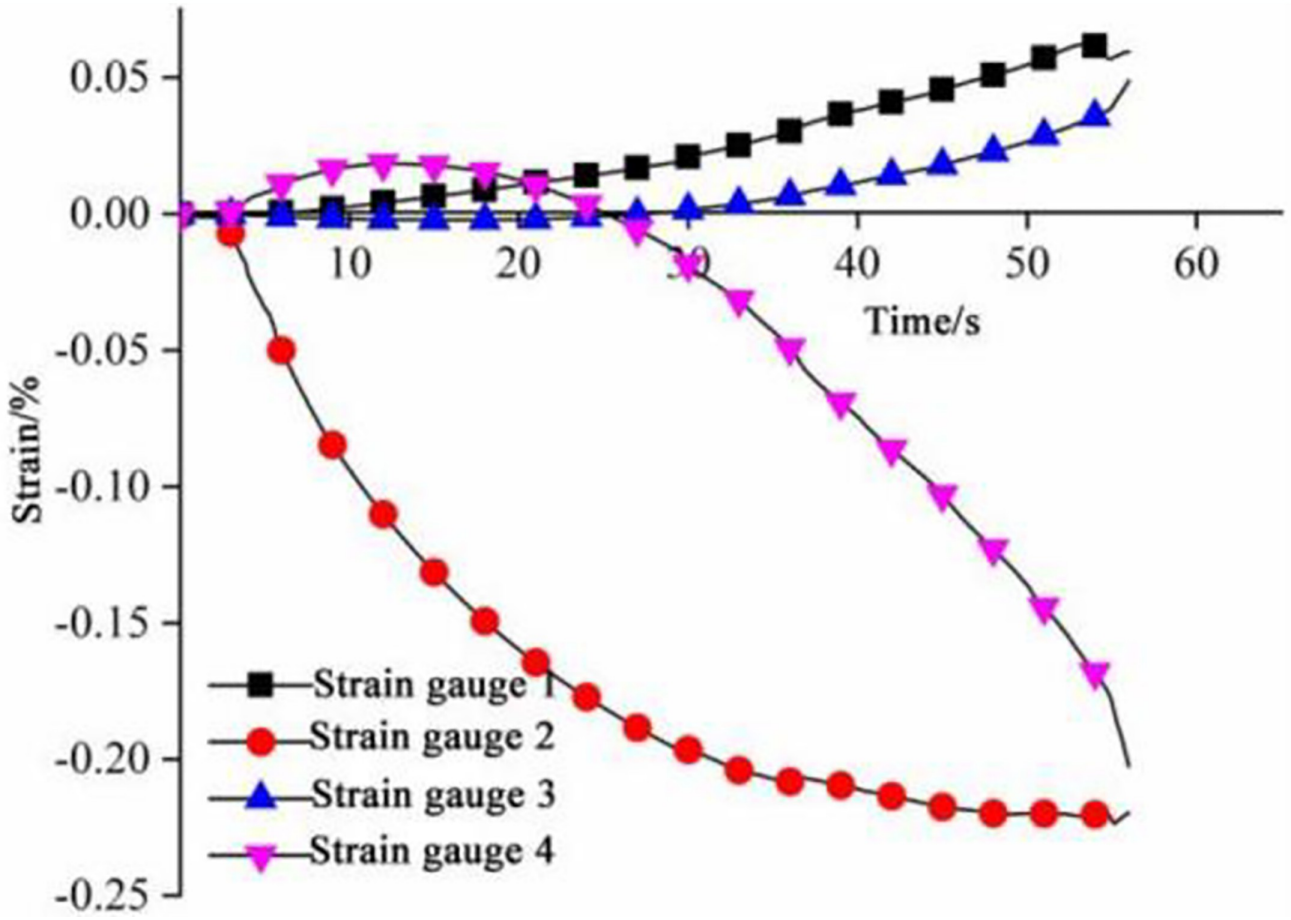
Strain at different positions of the sample.

After processing the collected AE wave data and strain data, the received AE waves and changes in strain at different positions of the sample were obtained as shown in Figs 15 and 16. Four channels 1, 2, 3, and 4 were used in the test. The AE waves in channels 1 and 3 arrive first and almost simultaneously, followed by the almost simultaneous arrival of AE waves in channels 2 and 4, as shown in Fig 15. Results indicate that the upper part of the sample is stressed and deformed first under compression, then the stress and deformation gradually propagate throughout the sample.

The AE cracking events begin to decrease within 20% of the peak load, when the AE count and energy are both low. The loading stage of the sample changes from development to the compression and compaction of microcracks. The energy produced by microcracking provides AE sources in the stage.

The stress gradually rises as the applied load approaches 40%, and correspondingly the AE count and energy also slowly increase. The sample undergoes elastic deformation with numerous microcracks forming therein, which further develop and propagate, leading to uniform failure of the sample.

When reaching 50% of the peak load, the waves develop rapidly, and the AE events increase significantly. Due to appearance of cracks, obvious stress concentration occurs in the stage, cracks begin to develop very non-uniformly, and ruptures appear in some weak regions. Changes in cracks inside the sample are shown as propagation and coalescence, and these cracks develop into macrocracks. As a result, damages occur in local regions, accompanied by active AE events.

When reaching 90% of the peak load, that is, at the brink of failure, AE events gradually decrease, forming a stage of relatively stable AE events. During failure of the sample, the AE events increase abruptly, and at the same time, numerous cracks coalesce in the sample. These coalescing cracks form a surface fracture, and finally the sample becomes unbalanced and is damaged. This final stage lasts for a rather short time period (~5 s) from beginning to ending, during which there is also the maximum AE count and peak AE energy. The sample does not burst apart after failure, and the maximum energy curve under load always shows a gentle descent and tends to be flat after decreasing to a certain level. In the gentle descent stage of the maximum energy curve, all relevant AE energy can be measured.

Compressive strain is found at load bearing points 1# and 3# while tensile strain at points 2# and 4# as shown in Fig 16. The strain at points 1# and 3# changes at a consistent rate, and the strain at point 1# is slightly larger than that at 3#; the strain rate increases at first and then decreases at point 2#; strain rate at point 4# decreases at first and then increases, indicating that the compressive stress is dominant in the longitudinal direction while tensile stress dominant in the transverse direction of the sample. The maximum longitudinal and transverse displacements are 0.045 mm and 0.25 mm, respectively. Therefore, the sample shows a failure mode characterized by longitudinal tearing. The strain rates change at the 35th s at strain gauges 2# and 4#, when the stress reaches the peak, followed by large deformation until failure of the sample.

## 4. Discussion

Although many scholars have made significant advancements in the in-depth study of the mechanical properties of various frozen soft rocks, research on the mechanical properties and strength standards of these materials remains limited. This paper presents undrained uniaxial and triaxial shear tests, as well as creep tests and acoustic emission tests conducted on soft rock under different confining pressures and freezing temperatures. The strength criterion and creep law for frozen soft rock were established, contributing valuable insights to the field. However, it is important to acknowledge the limitations of this study. Specifically, this research only considers the confining pressure and freezing temperature of the rock, while other influencing factors, such as initial water content and mineral composition, have not been addressed. Future studies will incorporate these factors for a more comprehensive understanding.

## 5. Conclusions

The following conclusions are drawn by performing uniaxial and triaxial tests as well as AE tests on frozen soft rocks at different temperatures under different confining pressures:

1. The uniaxial compressive strength first increases linearly and then slowly grows with the incremental strain. Finally, the stress drops suddenly, indicating brittle failure and strain softening characteristics.
2. The three-level uniaxial creep curve of soft rocks is shown as an attenuated stable creep curve overall, with an unclear first stage of creep.
3. Frozen soft rocks show brittle failure, and the stress—strain curves display softening characteristics. Under the same confining pressure, the failure strain of soft rock decreases with the decrease of temperature. The strength, elastic modulus, and Poisson’s ratio of the frozen soft rocks at -10°C are 17.5 MPa, 85 MPa, and 0.25, respectively. The failure process of the frozen soft rocks can be described using the M-C criterion, with a correlation coefficient greater than 0.98.
4. AE tests further verify that the soft rock sample exhibits shear failure under load, with the shear plane having an angle of 45° with the horizontal.

## Supporting information

S1 Table (XLSX)
